# A cluster randomized trial evaluating electronic prescribing in an ambulatory care setting

**DOI:** 10.1186/1745-6215-8-28

**Published:** 2007-10-03

**Authors:** Merrick F Zwarenstein, Katie N Dainty, Sherman Quan, Alex Kiss, Neill KJ Adhikari

**Affiliations:** 1 Senior Scientist, Institute For Clinical Evaluative Sciences and The Clinical Epidemiology Unit, Center For Health Services Sciences, Sunnybrook Hospital, 2075 Bayview Avenue, Toronto, Ontario, Canada; 2 Senior Scientist, Keenan Research Centre In The Li Ka Shing Knowledge Institute, 80 Bond Street, Toronto, Ontario, Canada; 3 Department of Medicine, Sunnybrook Health Sciences Centre, 2075 – C831 Bayview Avenue, Toronto, Ontario, Canada; 4 Department of Research Design & Biostatistics, Institute for Clinical Evaluative Sciences; 2075 Bayview Avenue, Toronto, Ontario, Canada; 5 Department of Critical Care Medicine, Sunnybrook Health Sciences Centre, 2075 Bayview Avenue, Toronto, Ontario, Canada; 6 Interdepartmental Division of Critical Care Medicine, University of Toronto, Toronto, Ontario, Canada

## Abstract

**Background:**

Medication errors, adverse drug events and potential adverse drug events are common and serious in terms of the harms and costs that they impose on the health system and those who use it. Errors resulting in preventable adverse drug events have been shown to occur most often at the stages of ordering and administration. This paper describes the protocol for a pragmatic trial of electronic prescribing to reduce prescription error. The trial was designed to overcome the limitations associated with traditional study design.

**Design:**

This study was designed as a 65-week, cluster randomized, parallel study.

**Methods:**

The trial was conducted within ambulatory outpatient clinics in an academic tertiary care centre in Ontario, Canada. The electronic prescribing software for the study is a Canadian electronic prescribing software package which provides physician prescription entry with decision support at the point of care. Using a handheld computer (PDA) the physician selects medications using an error minimising menu-based pick list from a comprehensive drug database, create specific prescription instructions and then transmit the prescription directly and electronically to a participating pharmacy via facsimile or to the physician's printer using local area wireless technology. The unit of allocation and randomization is by 'week', i.e. the system is "on" or "off" according to the randomization scheme and the unit of analysis is the prescription, with adjustment for clustering of patients within practitioners.

**Discussion:**

This paper describes the protocol for a pragmatic cluster randomized trial of point-of-care electronic prescribing, which was specifically designed to overcome the limitations associated with traditional study design.

**Trial Registration:**

This trial has been registered with clinicaltrials.gov (ID: NCT00252395)

## Background & rationale

A medication error, as defined by the National Coordinating Council for Medication Error Reporting and Prevention (NCCMERP), is a preventable event that may cause or lead to inappropriate medication use or patient harm. Such events may be related to practice, products, procedures, and systems, including prescribing, order communication, product labelling, packaging, nomenclature, compounding, dispensing, distribution, administration, education, monitoring, and use [1].

Medication errors, adverse drug events (ADEs), and potential adverse drug events impose substantial harms and costs on patients and the health system. The largest study [2] of the prevalence of ADEs and potential ADEs suggests rates of 6.5 ADEs and 5.5 potential ADEs per 100 hospital admissions. Of the life-threatening and serious ADEs in this study, 42% were preventable, compared with 18% of significant ADEs. Errors resulting in preventable ADEs occurred most often at the stages of ordering (56%) and administration (34%); transcription (6%) and dispensing errors (4%) were less common.

There are little data available on errors (as distinct from ADEs, which reflect actual harm). In one study [3] of 36 participating inpatient institutions, 605 (19%) of 3216 medication doses were given in error. Seven percent of these errors were found to be potentially harmful to patients. However, this study was designed to capture only dispensing errors and not prescribing errors.

Changing clinical practices in the area of prescribing error has been widely discussed. In general, it is thought that systems changes rather than individual educational interventions will be required to trap errors, and the main solution widely discussed is the use of computerized physician order entry (CPOE).

There is reliable evidence that computerized order entry reduces ADEs, but the systems used to date have all been desktop-based. In a systematic review [4] of well-conducted evaluations (one of which was a randomized trial) of physician order entry systems and a recent Canadian randomized trial, the separate studies showed findings such as 81% decrease in total medication errors, 55% decrease in non-intercepted serious medication errors, 13% decrease in cases of inappropriate dosing and 24% decrease in inappropriate frequency of nephrotoxic drugs, all of which were statistically significant. These are substantial benefits, with a median of 55% improvement. These studies and their resultant benefits were obtained in the inpatient setting, where the sheer intensity of prescribing and complexity of patient condition could be expected to result in much higher levels of error. However, there is no reason to believe that the relative benefit obtained in a high intensity setting cannot also be obtained in a lower intensity one.

All reliably evaluated CPOE systems to date have been desktop computer based, but there is widespread use and acceptance among physicians of PDAs (personal digital assistants), in many roles. Clinicians report that drug information on a PDA improved their access to information and efficiency while reducing their self-perceived error rates [5]. The use of these devices for information retrieval in clinical settings is expected to grow as wireless communication becomes more ubiquitous and as more applications become available [5,6]. During the past decade there has been a growing awareness of several interventions, such as point of care information technology, aimed at promoting safer health care for our patients. As far as we are aware, our study is the first to rigorously evaluate PDA-based prescribing systems in which drug interaction, dosing alerts, and adverse event reporting are fully integrated.

This paper describes the protocol for a pragmatic cluster randomized trial of point-of-care electronic prescribing, which was specifically designed to overcome the limitations associated with traditional study design.

## Methods

This study was approved by both the participating hospital and the University of Toronto research ethics boards. A standing advisory committee provided input into the design and conduct of the study since inception and has continued to meet regularly during the implementation. This trial has also been registered with clinicaltrials.gov (identifier NCT00252395).

### The Intervention

A field-tested Canadian electronic prescribing software package (Drugmagnet, Toronto, Ontario) designed for physician prescription entry with decision support at the point of care was used for this study. A physician, using a battery powered PDA (Palm Pilot or equivalent) selects medications using an error minimising menu-based pick list from a comprehensive drug database, creates specific prescription instructions and then transmits the prescription directly and electronically to a participating pharmacy via facsimile or to the physician's printer using local area wireless technology. Each prescription order is checked against stored evidence-based guidelines and a dosing and interactions database.

### Study population

This trial has been conducted within ambulatory outpatient clinics at an academic health sciences centre in Toronto, Ontario, Canada (August 2005 – October 2006). Both a specialist setting (Rheumatology, Dermatology and Cardiology patients) and a primary care Family Medicine setting were used. There are approximately 40 full-time physicians within these clinics, who see a total of approximately 1000 patients each week.

### Study design

The study was a 65-week, cluster randomized (3:1 allocation ratio), parallel study with time as the element of randomization. The randomization scheme was computer generated by the biostatistics department at the hospital. The unit of allocation and randomization was the 'week'; although the unit of analysis is the prescription, with adjustment for clustering of patients within physicians. All participating physicians were trained to use electronic prescribing software, but only half of the weeks in the study period were allocated as intervention or "on" weeks, during which the software was switched on, and prescribing was carried out electronically.

Physician training consisted of a simple two hour training course and follow-up tutoring where necessary. The Health Informatician on the project used regular check-ins and a help-line pager to ensure that all technical issues were fixed in a timely manner and that any prevalent user errors with regard to the software were addressed. No further training was conducted in order simulate real-world conditions with limited user support.

During control periods all prescriptions were written on paper as per standard practice. During intervention periods or "on" weeks, all prescriptions were to be filled using the PDAs and the electronic prescribing software. The randomization was employed by turning the prescribing software servers in each clinic on and off according to the randomization scheme. A health informatics assistant was responsible for carrying out the randomization and the allocation was kept concealed until midnight on the last day of the previous week. If a physician tried to enter a prescription on his or her handheld on a control or "off" week, the system immediately gave a message that the wireless network was not been detected, and that the software was inaccessible.

### Outcomes

The principle outcome measure was total prescribing error, as measured by the number of call backs received from pharmacists to prescribing physicians offices. These are defined as potential medication errors picked up by the pharmacist at the time the prescription is filled. Rates will be compared between intervention and control weeks. Prescribing errors can take many forms and are of different levels of severity and a single prescription can have several errors and all will be counted. The denominator for total error is the total number of prescriptions and the numerator is the sum of the following types of error:

#### a) Interactions

These include errors in which interacting drugs are prescribed, and these interactions can vary between minor and major. Four standard levels are described- severe, in which harm is likely; moderate, in which harm may occur in some situations, but it is reasonable for the physician to prescribe the drug provided that special monitoring or other interventions are simultaneously indicated; potential drug interactions in which an interaction is theoretically possible, but unlikely or likely but of trivial importance; and extremely rare potential interaction. All of the above will be counted in the data analysis.

#### b) Dosing and route of administration errors

These are errors in which the prescribed dose or route of administration are incompatible with the recommended guidelines for that patient, based on weight, concurrent drugs or illnesses, and age.

#### c) Legibility

These are errors in which the writing on the prescription is not easily legible, in the pharmacist's opinion.

### Sample size and analysis plan

The unit of analysis for this trial is the individual prescription, adjusting for clustering of patients within physicians. Given an estimated intra-class correlation value of 0.05, a minimum of 65 weeks of prescribing is required to have 80% power for a two sided test to detect an absolute decrease of 5% from with a control baseline error rate of 25% with a type I error of 5%.

Descriptive statistics will be calculated for all variables of interest. Continuous measures will be summarized using means and standard deviations whereas categorical measures will be summarized using counts and percentages. The principal outcome measure, total prescribing error using PDA-generated versus written prescriptions, will be assessed using a two sample, two-sided test of proportions. This analysis will be performed on an intention-to-treat basis. All analyses will be carried out using the SAS Version 9.1 statistical program (SAS Institute, Cary, NC, USA).

### Data collection

Data on prescription errors was captured in two formats. One set of data consisted of data points recorded on a form during callbacks to the participating physicians' offices from pharmacies regarding prescription errors. A second set of data was derived from forms completed by the hospital internal pharmacy for each prescription filled there. These forms also include indicators related to prescribing errors and legibility. Data forms were collected weekly and transcribed into a database for analysis.

To guarantee privacy and confidentiality, all research data were kept within the hospital by professional staff operating under codes of conduct and using encrypted data capture systems. All patient and prescription data were anonymized after linkage. The electronic prescribing technology uses the highest existing levels of data encryption and its mode of operation conforms to professional obligations on confidentiality and Canadian and Ontario privacy laws.

## Discussion

This trial will be able to evaluate electronic prescribing in a hospital setting effectively while preserving the benefits of randomization to reduce bias. Its design takes into account the need for internal and external validity, that the results are attributable to the intervention and that they may be applied to general practice. This design is practical and does not interfere with usual physician workflow.

The findings from this trial will provide important information about the role of electronic prescribing in the ambulatory setting for clinicians and healthcare policy-makers in distinguishing.

One of the key decisions that required consideration in the design of this trial was the question of the appropriateness of physician- or cluster-level randomization. Rather than allocating physicians between the two groups using a random process, cluster-level randomized trials allocate groups of participants. In this study we randomized by time periods. The rationale for this randomization scheme is apparent when there is a high risk of heterogeneity between groups and to allow for the participating physicians to serve as their own controls groups.

This study will be the first published randomized control trial designed to evaluate the effectiveness of electronic prescribing in reducing prescription error in an ambulatory setting. Although technology applications related to patient safety and human error reduction are sweeping the healthcare landscape, it is important that we do not fall in to the same trap as our forefathers did with medications and snake oils. It is as important if not imperative to rigorously evaluate these applications in pragmatic, real-world fashion so that we can determine if they are truly effective or more of a hindrance than help. We look forward to adding the results of this trial to the growing body of literature in this area.

## Abbreviations

ADE – Adverse drug event

CPOE – Computerized physician order entry

PDA – Personal digital assistant

## Competing interests

The author(s) declare that they have no competing interests.

## Authors' contributions

MFZ (Primary Investigator) was responsible for the study design, randomization and analysis and interpretation of the data; has been directly involved in drafting the manuscript and critical review of the content; and has given final approval of the version to be published.

KND (Co-investigator) was involved in the study design, implementation of the project, acquisition of the data and analysis and interpretation of the data; has been directly involved in drafting the manuscript and critical review of the content; and has given final approval of the version to be published.

SQ (Co-investigator) was involved in the concept and design of the study, implementation of the project, acquisition of the data; has been directly involved in critical review of the manuscript content; and has given final approval of the version to be published.

AK (Biostatistician) was responsible for the study design, randomization and analysis and interpretation of the data; has been directly involved in critical review of the manuscript content; and has given final approval of the version to be published.

NKJA (Co-Investigator) was responsible for concept and study design and analysis and interpretation of the data; has been directly involved in drafting the manuscript and critical review of the content; and has given final approval of the version to be published.
